# THE LEGACY OF HELEN KIVNICK: THE IMPACT OF VITAL INVOLVEMENT AND CREATIVITIES ON WELL-BEING IN LATE LIFE

**DOI:** 10.1093/geroni/igac059.1482

**Published:** 2022-12-20

**Authors:** Carolyn Adams-Price

## Abstract

Helen Kivnick was an extraordinary woman whose body of work strongly influenced and continues to influence the field of gerontology. Her classic work on vital involvement in late life, some of which was conducted with Erik and Joan Erikson, could be considered the foundation of current perspectives on positive gerontology, and creativity and aging. The current symposium will include both personal and theoretical takes on the legacy of Kivnick’s work, especially her interests in vital involvement and creativity. Kivnick emphasized the possibility for older adults to adapt to new situations, even in very late life, and while experiencing physical and cognitive decline. Two of the speakers will speak on their personal experiences with Kivnick, while two others will emphasize research and theory derived from Kivnick’s work. Dr. Adams-Price will introduce the symposium by addressing the significance of vital involvement as a concept in aging, as well as the ways creativity can improve well-being in late life. Dr. Wyatt-Brown will describe her long friendship with Dr. Kivnick, including Kivnick’s comments in their book with Ruth Karpen and Margaret Gullette. The Big Move, which describe Wyatt-Brown’s life with her husband in continuing care. Dr. Chee will discuss her research on patients with dementia who participated in a story-telling intervention, which increased their meaningful engagement in their environment. Finally, Ms. Linda Davis will recall her long partnership with Kivnick and the results of the national study they conducted on the benefits of creativity interventions for at risk- older adults in HUD housing.

